# Supplementary material for: Regulation of species metabolism in synthetic community systems by environmental pH oscillations
Source: Nat Commun. 2023 Nov 18;14:7507. doi: 10.1038/s41467-023-43398-6 (PMC10657449; doi:10.1038/s41467-023-43398-6)
Supplement: Supplementary file 1 — Supplementary Information [file 41467_2023_43398_MOESM1_ESM.pdf]

Regulation of species metabolism in synthetic community systems by environmental pH oscillations

Li *et al.*

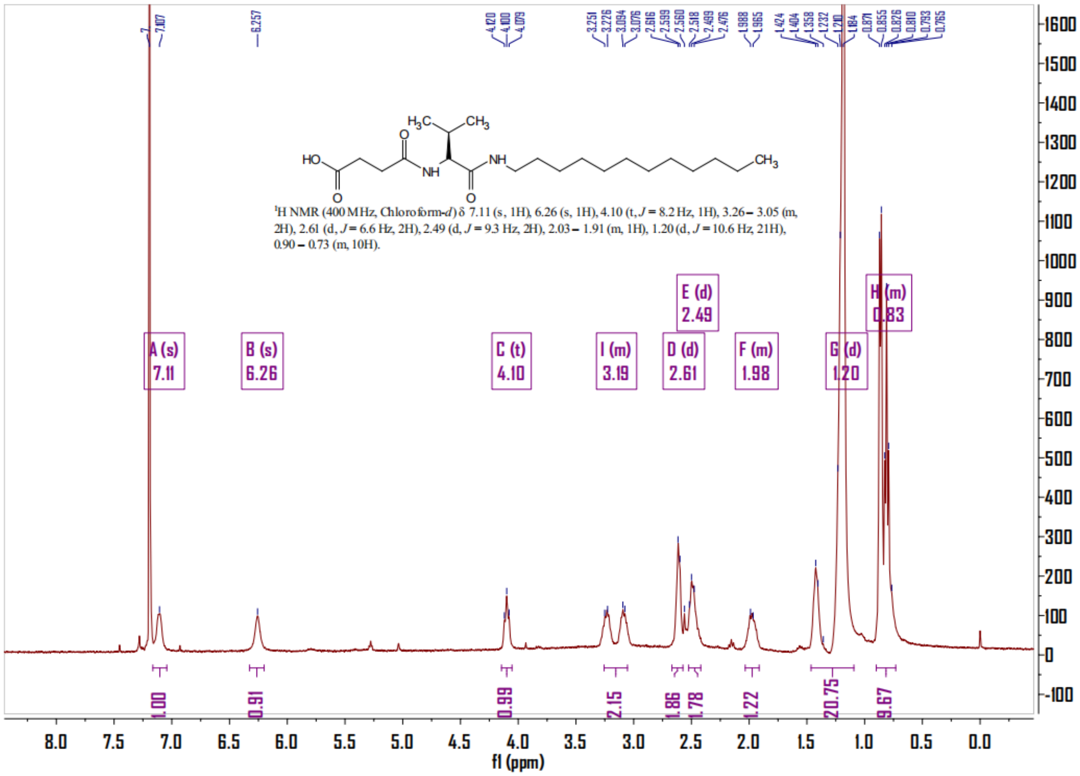

Supplementary Fig. 1. The ^{1}H NMR spectrum of pH-responsive molecule.

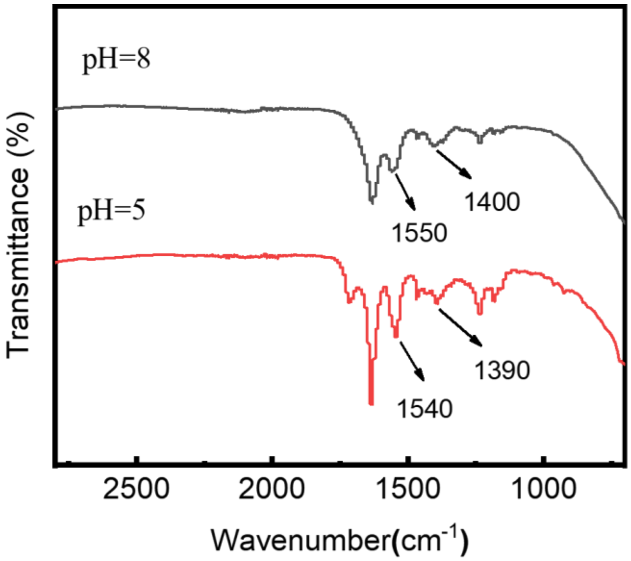

Supplementary Fig. 2. ATR-FTIR spectra at different pH conditions. Source data are provided as a Source Data file.

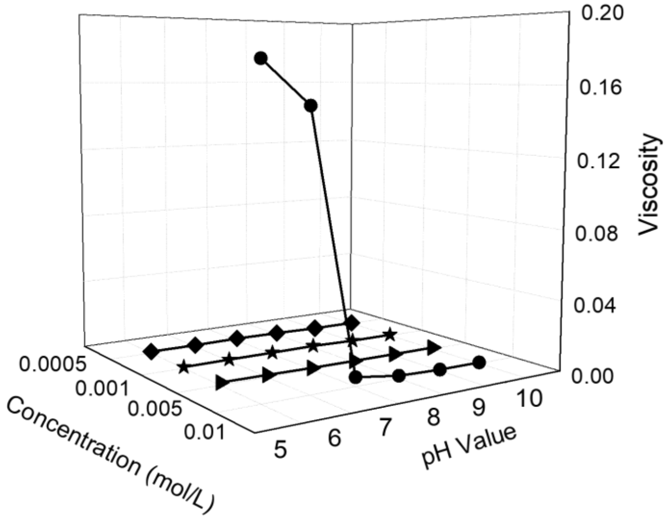

Supplementary Fig. 3. The inner viscosity of artificial cells containing pH-responsive-molecule as a function of its concentration and pH value measured using single particle tracking technique. Source data are provided as a Source Data file.

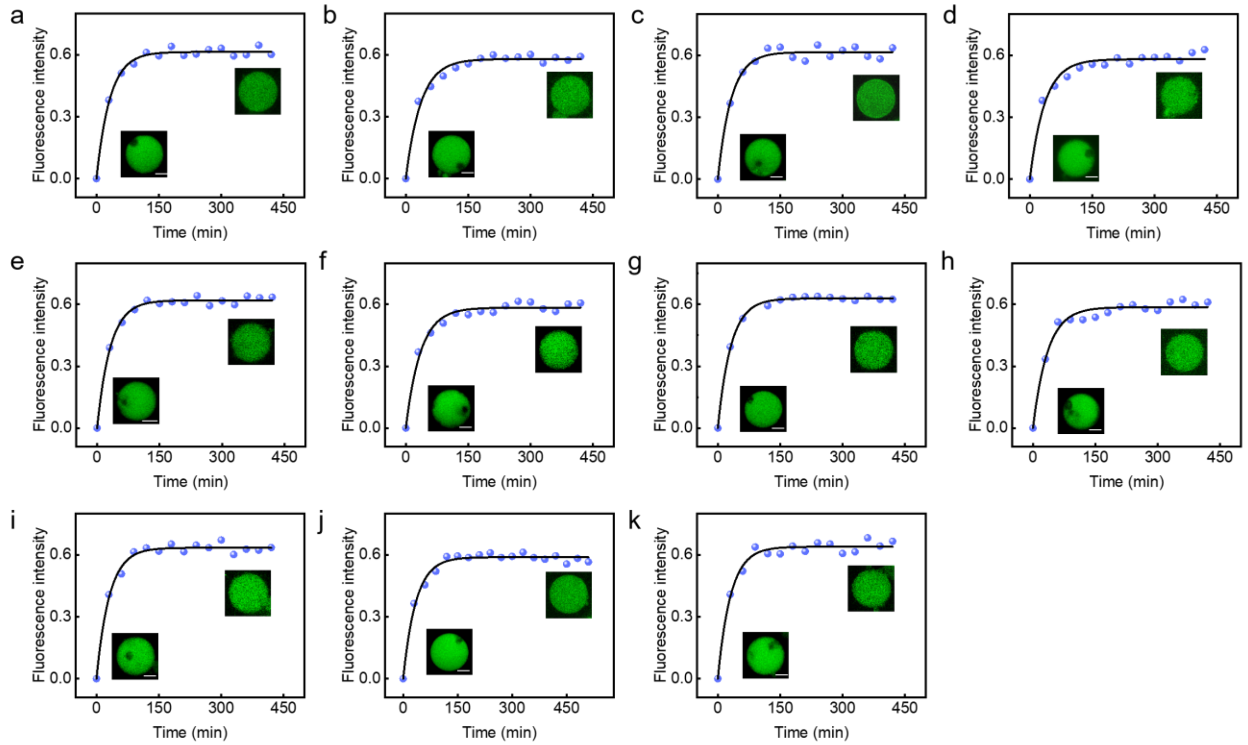

Supplementary Fig. 4. Fluorescence recovery after photobleaching data of artificial cells containing pH-responsive molecule (0.01 mol/L) and FITC-BSA (3.7 μM). pH value of 6.5 (**b, d, f, h, j**) and 6.6 (**a, c, e, g, i, k**). Scale bar was 2 μm. Source data are provided as a Source Data file.

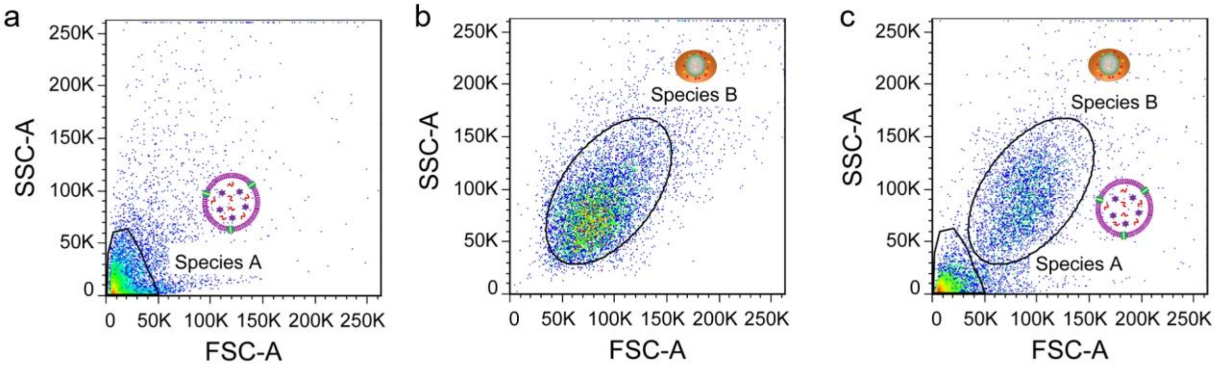

Supplementary Fig. 5. FACS-derived 2D dot plots of side-scattered light (SSC) vs. forward-scattered light (FSC). Species A (**a**), Species B (**b**), two-species-community (**c**). Total number of particles counted was 10,000. Species A and species B were pH-responsive artificial cells containing sucrose and *Saccharomyces cerevisiae*, respectively.

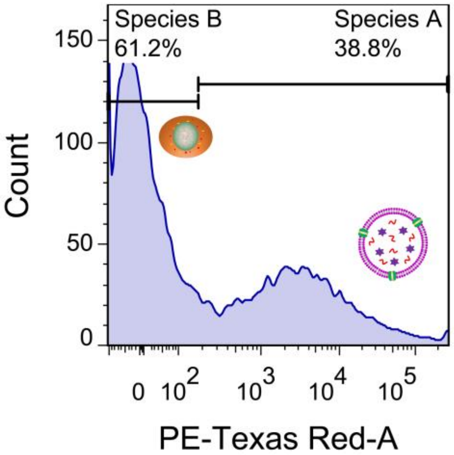

Supplementary Fig. 6. Flow cytometry histograms of species B and A in a two-species-community. Species A were labeled with Texas Red-PE, while species B were not labeled.

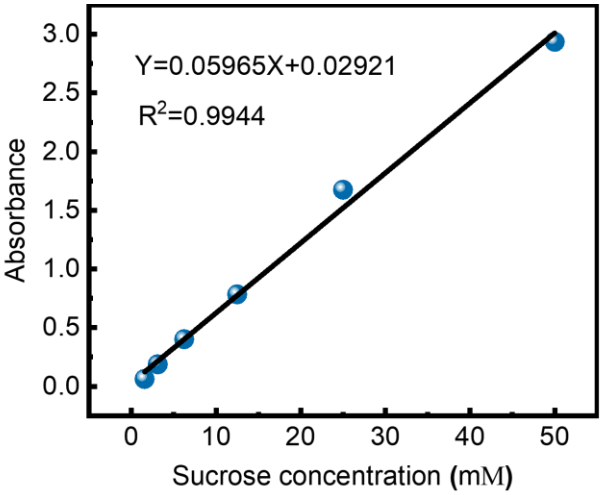

Supplementary Fig. 7. The calibration curve of sucrose obtained from the sucrose detection kit. Source data are provided as a Source Data file.

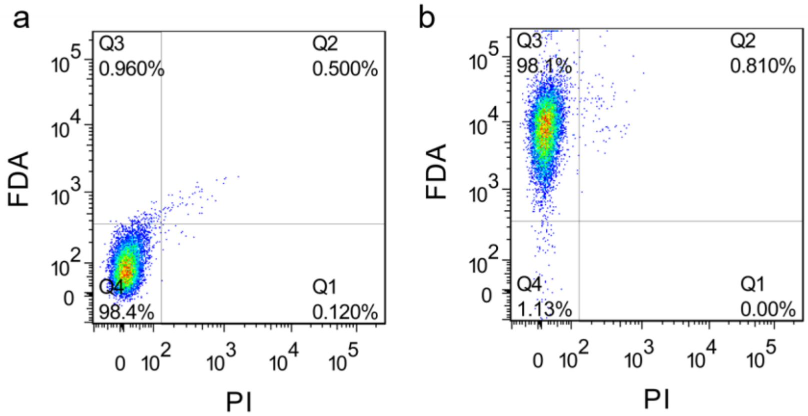

Supplementary Fig. 8. Flow cytometry scatter plots of species B cultured with 41 mM sucrose. (**a**) Flow cytometry scatter plots of nonlabelled species B. (**b**) Flow cytometry scatter plots of live-dead stained species B at initial time (0 min). Live *Saccharomyces cerevisiae* (species B) was stained with FDA (green, in Q3), and dead *Saccharomyces cerevisiae* was stained with PI (red, in Q1). Total number of particles counted, 10,000. Species B was *Saccharomyces cerevisiae*.

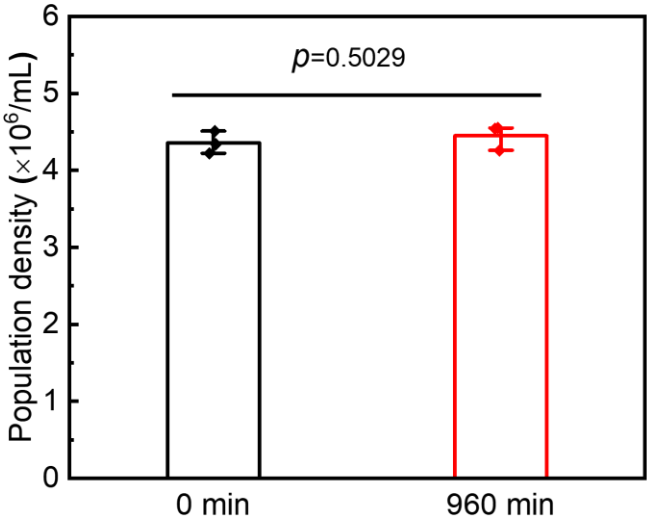

Supplementary Fig. 9. The species B population density at initial time (0 min) and at longest oscillation time (960 min) cultured in 41 mM sucrose solution. Species B was *Saccharomyces cerevisiae*. The population density of species B is obtained from three independent samples. Data are presented as the mean values ± SDs, n=3. Statistical analyses were carried out by two-tailed unpaired student's t-test. Degrees of freedom = 4, p = 0.5029, effect size statistic = 0.6004, Confidence Intervals = 95%. Source data are provided as a Source Data file.

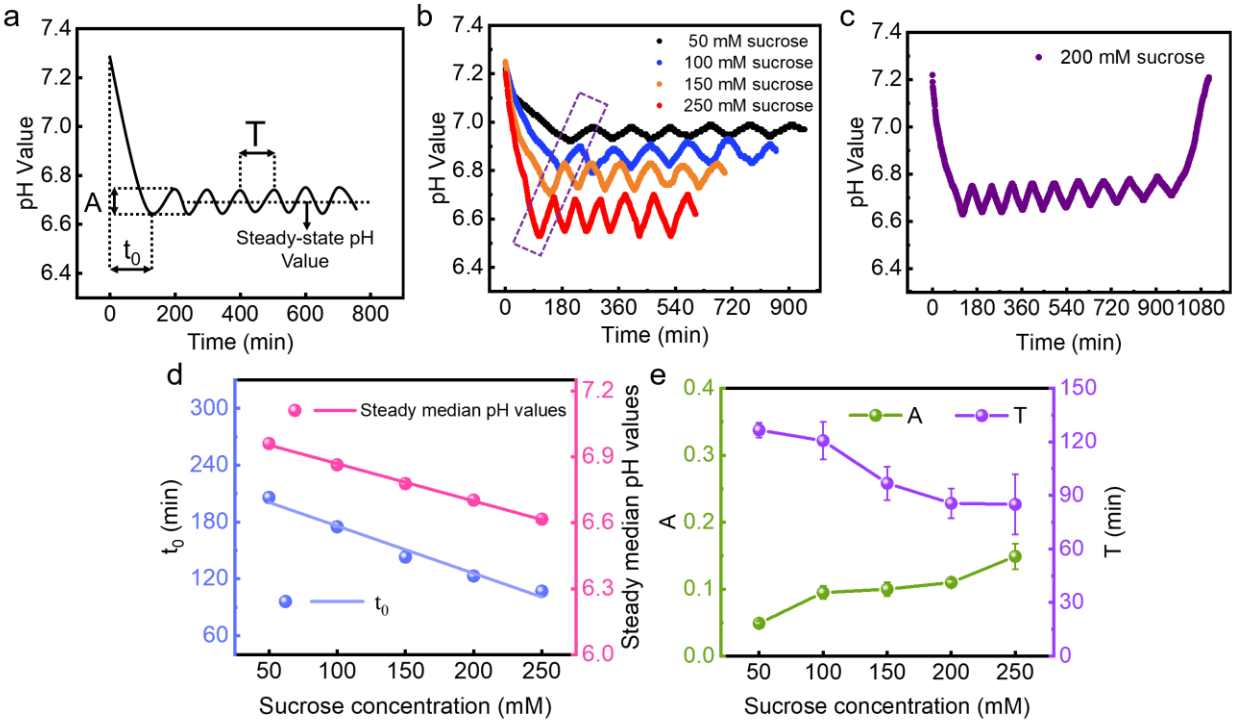

Supplementary Fig. 10. The solution pH oscillation of two-species-community system with species A containing different sucrose concentrations. (**a**) Schematic diagram of pH oscillation to define oscillation parameters. (**b**) The solution pH oscillation of two-species-community system as a function of time with different sucrose concentration in species A. (**c**) The solution pH oscillation of two-species-community system as a function of time with 200 mM sucrose concentration in species A. (**d**) t_0 and the steady median pH value as a function of sucrose concentration in species A. (**e**) A and T as a function of sucrose concentration in species A. The average of A is from ten independent samples. Data are presented as the mean values ± SDs, n=10. The average of T is from five independent samples. Data are presented as the mean values ± SDs, n=5. Source data are provided as a Source Data file.

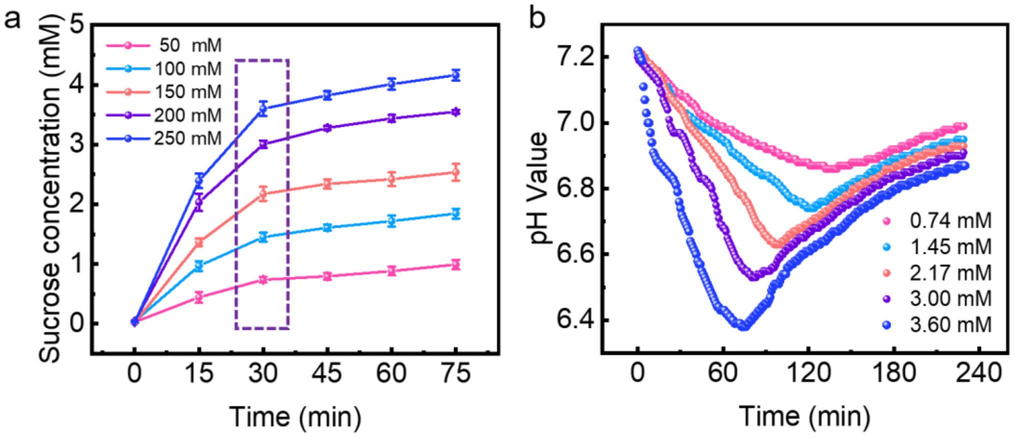

Supplementary Fig. 11. The sucrose leaking behavior from species A and solution pH variation by mixing *Saccharomyces cerevisiae* and sucrose. (**a**) The sucrose concentration leaking out from species A (encapsulating different initial sucrose concentrations) as a function of time. The concentration of sucrose is obtained from three independent samples. Data are presented as the mean values ± SDs, *n*=3. (**b**) The solution pH variation over time caused by the mixing of *Saccharomyces cerevisiae* and sucrose with the concentration after 30 minutes leakage from species A (purple box in **a**). The sucrose concentration leaking out for 30 minutes from species A containing 50 mM, 100 mM, 150 mM, 200 mM, 250 mM are 0.74 ± 0.05 mM, 1.45 ± 0.08 mM, 2.17 ± 0.12 mM, 3 ± 0.06 mM, and 3.6 ± 0.12 mM, respectively. Source data are provided as a Source Data file.

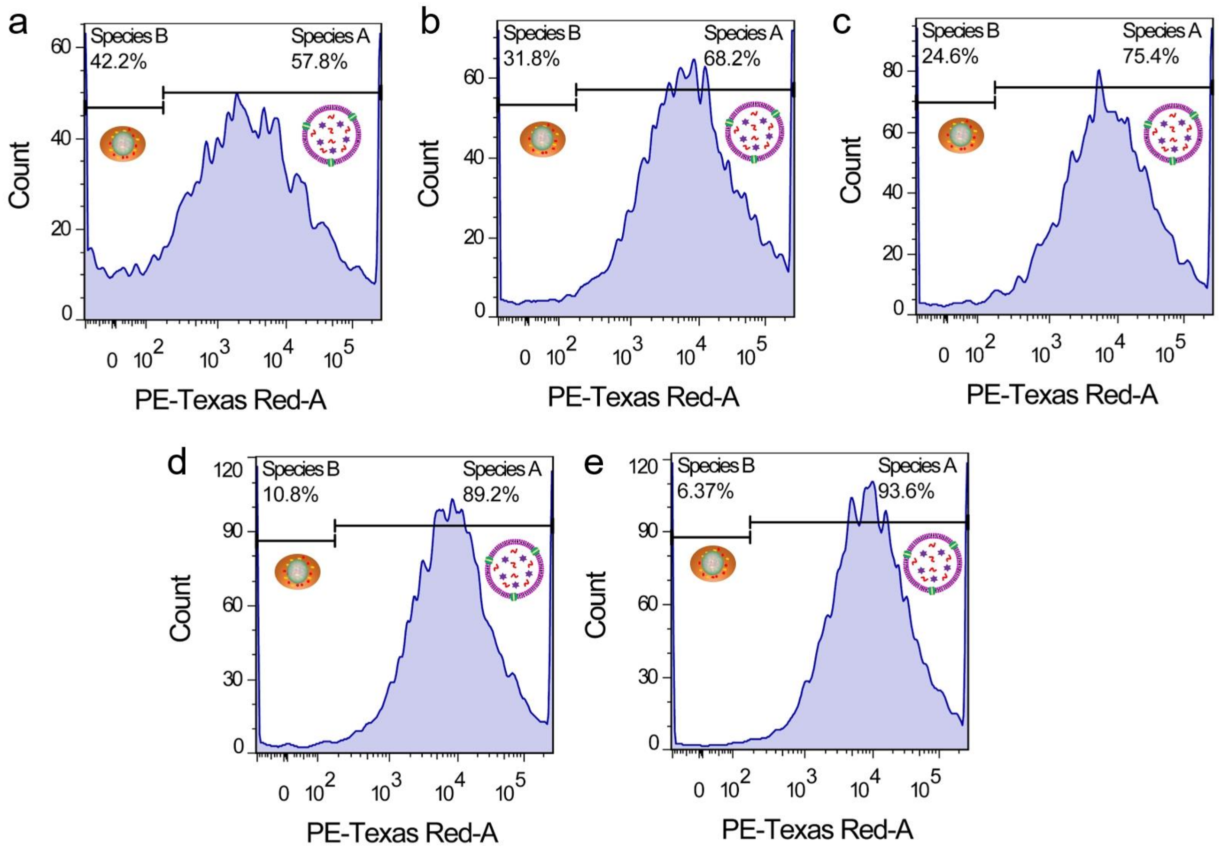

Supplementary Fig. 12. Flow cytometry histograms of species A and B in a two-species-community. Ratio of species A to B of 1.4:1 (**a**), 2.1:1 (**b**), 3.1:1 (**c**), 8.3:1 (**d**), 15.0:1 (**e**), respectively. Total number of particles counted was 10,000. Species A were labeled with Texas Red PE, while species B were not labeled. Species A and species B were pH-responsive artificial cells containing sucrose and *Saccharomyces cerevisiae*, respectively.

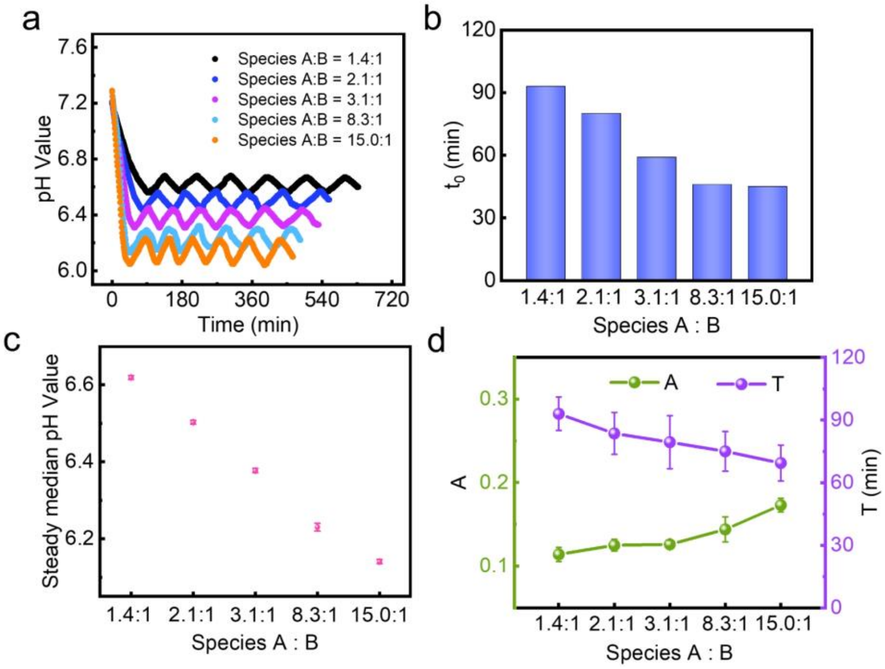

Supplementary Fig. 13. The solution pH oscillation of two-species-community system with different species ratios. (**a**) The solution pH oscillation of two-species-community system as a function of time with different ratio of species A to B. (**b**) t_0 as a function of ratio of species A to *B*. (**c**) The steady median pH value as a function of ratio of species A to B. The average of steady median pH value is from ten independent samples. Data are presented as the mean values ± SDs, n=10. (**d**) A and T as a function of ratio of species A to B. The average of A is from ten independent samples. Data are presented as the mean values ± SDs, n=10. The average of T is from five independent samples. Data are presented as the mean values ± SDs, n=5. Species A and species B were pH-responsive artificial cells containing sucrose and *Saccharomyces cerevisiae*, respectively. Source data are provided as a Source Data file.

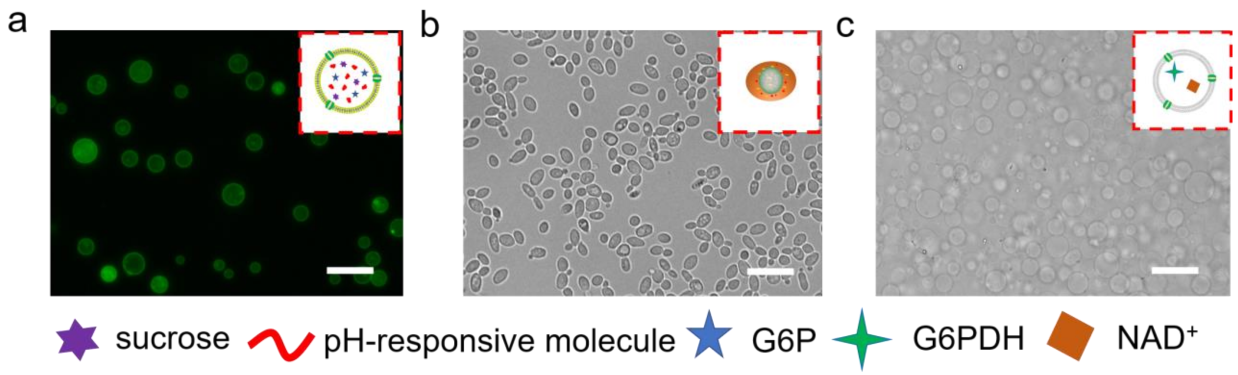

Supplementary Fig. 14. Microscopy images of species A′, B, and C. Fluorescence image of species A′ labeled with NBD-PE (green) (**a**), and bright field images of nonlabelled species B (**b**) and species C (**c**). Scale bar = 20 μm. Species A′, species B and species C were pH-responsive artificial cells containing sucrose and G6P, *Saccharomyces cerevisiae* and artificial cells containing NAD^+ and G6PDH, respectively. G6PDH and G6P are the abbreviations of glucose-6-phosphate dehydrogenase and glucose-6-phosphate, respectively. NAD^+ is the abbreviations of nicotinamide adenine dinucleotide.

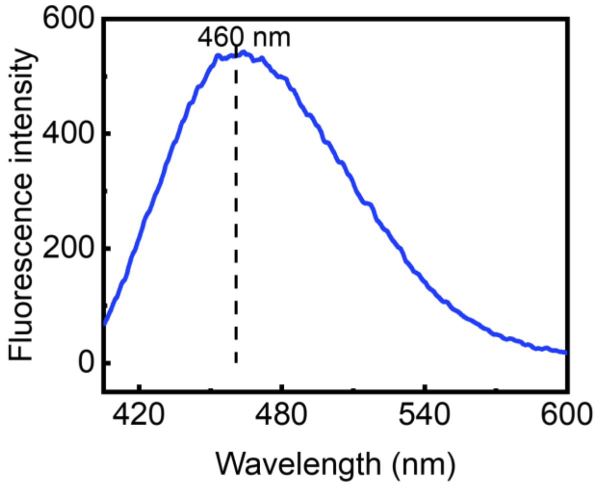

Supplementary Fig. 15. The fluorescence spectrum of NADH. NADH is the abbreviations of nicotinamide adenine dinucleotide plus hydrogen. Source data are provided as a Source Data file.

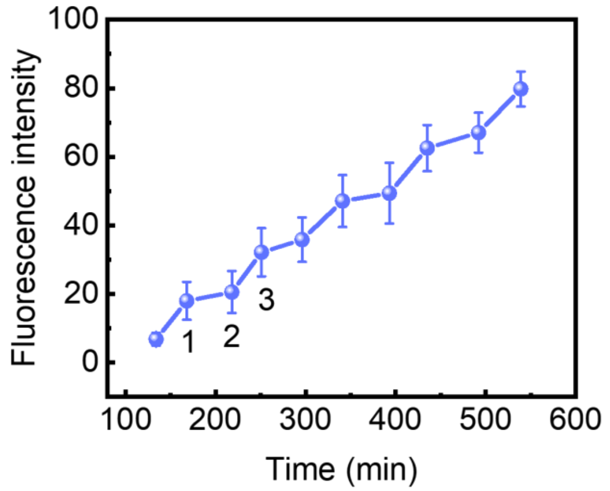

Supplementary Fig. 16. The average fluorescence intensity in species C at different oscillating point corresponding to Fig 3d. Data are presented as mean values ± SD, n = 2. Species C was artificial cells containing NAD^+ and G6PDH. Source data are provided as a Source Data file.

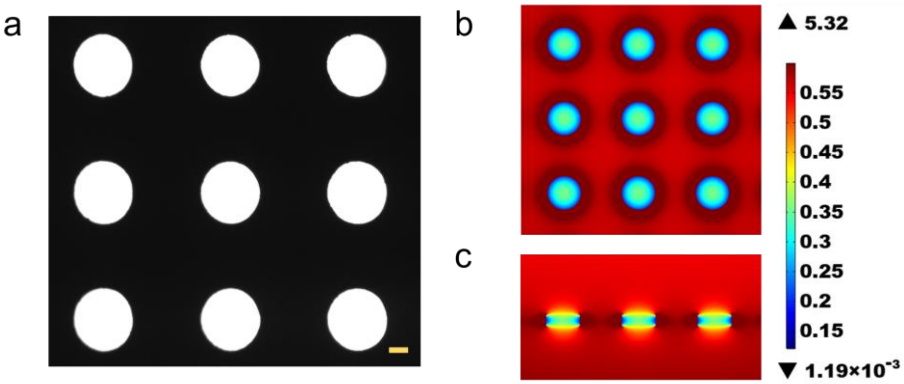

Supplementary Fig. 17. Microscopy image of stainless-steel mesh and its magnetic field simulation. (**a**) Bright field image of the stainless-steel mesh with microwell diameter of 300 μm. The scale bar was 100 μm. (**b**) Simulation results of the magnetic field distribution inside microwells. (**c**) Simulation results of the magnetic field distribution profile of the cross section of microwells. Dark blue areas indicated the weak magnetic field regions.

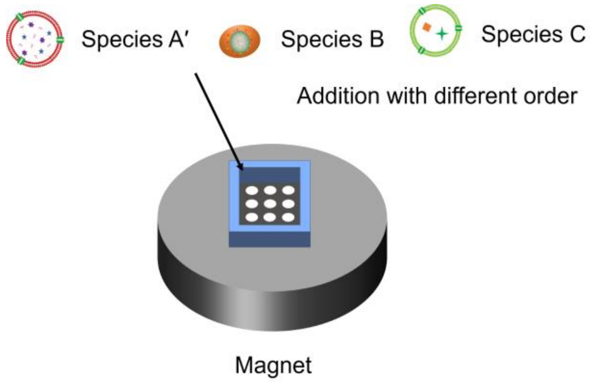

Supplementary Fig. 18. Schematic illustration of the home-made device for spatially coded three-species-community using Magneto-Archimedes principle. Species A′, species B and species C were pH-responsive artificial cells containing sucrose and G6P, *Saccharomyces cerevisiae* and artificial cells containing NAD^+ and G6PDH, respectively.

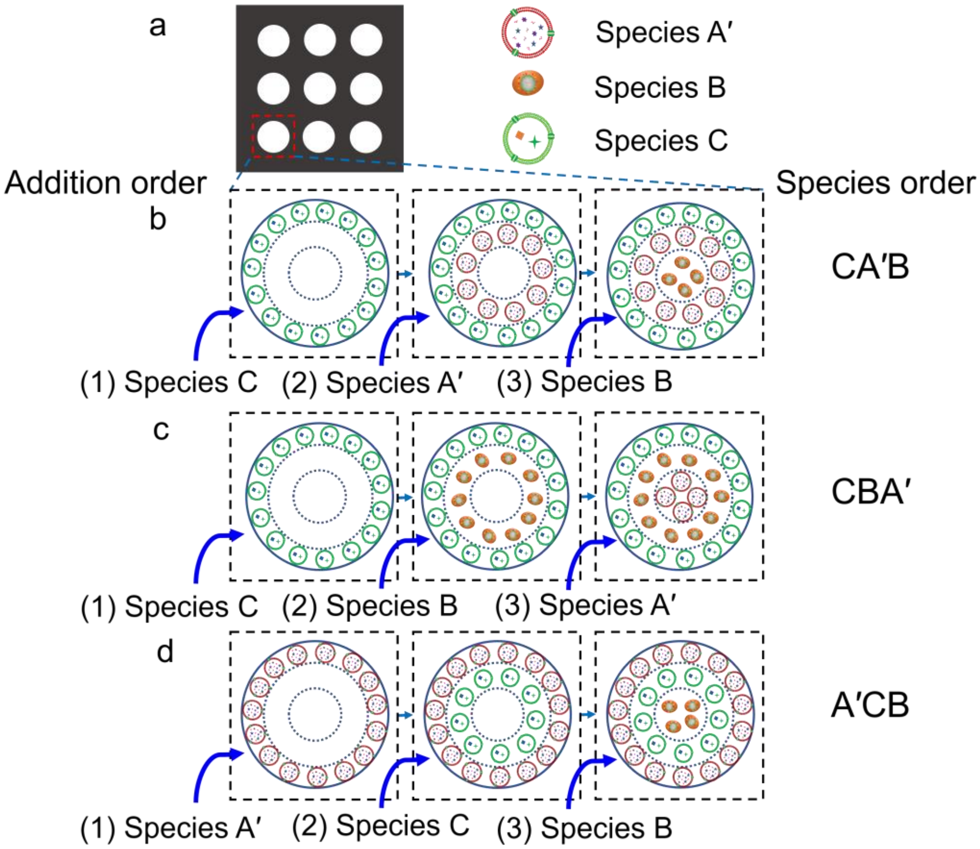

Supplementary Fig. 19. The schematic diagram of the preparation of spatially coded three-species-communities. (**a**) The schematic diagram of stainless steel mesh. (**b**) The schematic diagram of the preparation of CA′B community by sequentially adding species C, species A′ and species B into holes of (**a**). (**c**) The schematic diagram of the preparation of CBA′ community by sequentially adding species C, species B and species A′ into holes of (**a**). (**d**) The schematic diagram of the preparation of A′CB community by sequentially adding species A′, species C and species B into holes of (**a**). Species A′, species B and species C were pH-responsive artificial cells containing sucrose and G6P, *Saccharomyces cerevisiae* and artificial cells containing NAD^+ and G6PDH, respectively.

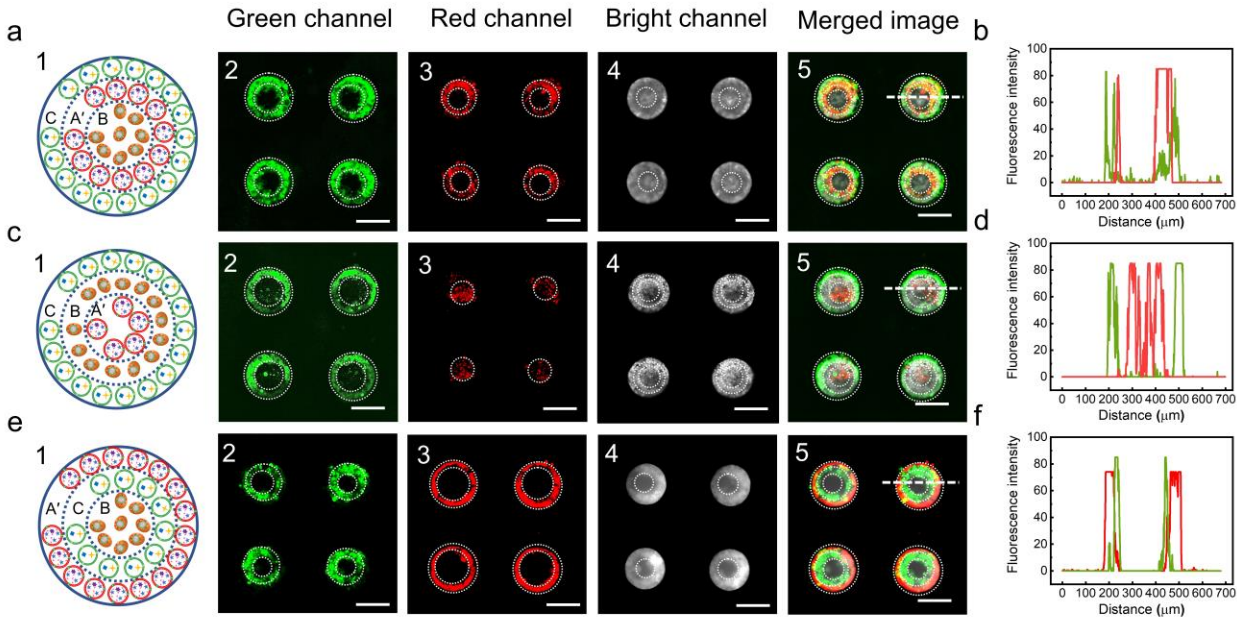

Supplementary Fig. 20. The schematic diagram and microscopy images of spatially coded three-species-communities. Construction of spatially coded three-species-community arrays of CA′B (**a**), CBA′ (**c**) and A′CB (**e**). Species A′, B, and C were artificial cells containing sucrose and G6P (red color, labelled with Texas Red-PE), *Saccharomyces cerevisiae*, and artificial cells containing NAD^+ (green color, labelled with NBD-PE), respectively. Schematic (**a1**) and fluorescence images of CA′B with green channel (**a2**), red channel (**a3**), bright field (**a4**), and their merged image (**a5**). (**b**) The fluorescence profile of the white dashed line in (**a5**). Schematic (**c1**) and fluorescence images of CBA′ with green channel (**c2**), red channel (**c3**), bright field (**c4**), and their merged image (**c5**). (**d**) The fluorescence profile on the white dashed line in (**c5**). Schematic (**e1**) and fluorescence images of A′CB with green channel (**e2**), red channel (**e3**), bright field (**e4**), and their merged image (**e5**). (**f**) The fluorescence profile of the white dashed line in (**e5**). Scale bares were 200 μm. Species A′, species B and species C were pH-responsive artificial cells containing sucrose and G6P, *Saccharomyces cerevisiae* and artificial cells containing NAD^+ and G6PDH, respectively. Source data are provided as a Source Data file.

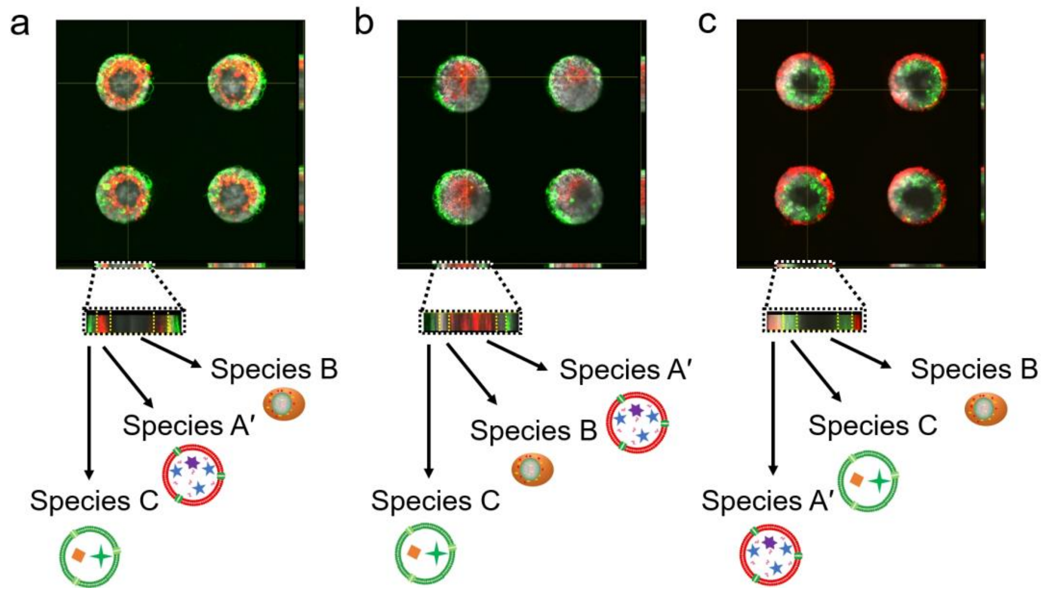

Supplementary Fig. 21. The projected laser scanning confocal images of the spatially coded three-species-community. CA′B (**a**), CBA′ (**b**) and A′CB (**c**). The order of species C (green), species A′ (red) and species B (gray) from left to right in the dotted line box in zoom-in image of (**a**) proved the spatially order of the community of CA′B. The order of species C (green), species B (gray) and species A′ (red) from left to right in the dotted line box in zoom-in image of (**b**) proved the spatial order of the community of CBA′. The order of Species A′ (red), species C (green) and species B (gray) from left to right in the dotted line box in zoom-in image of (**c**) proved the spatial order of the community of A′CB. Scale bars were 200 μm. Species A′, species B and species C were pH-responsive artificial cells containing sucrose and G6P, *Saccharomyces cerevisiae* and artificial cells containing NAD^+ and G6PDH, respectively.

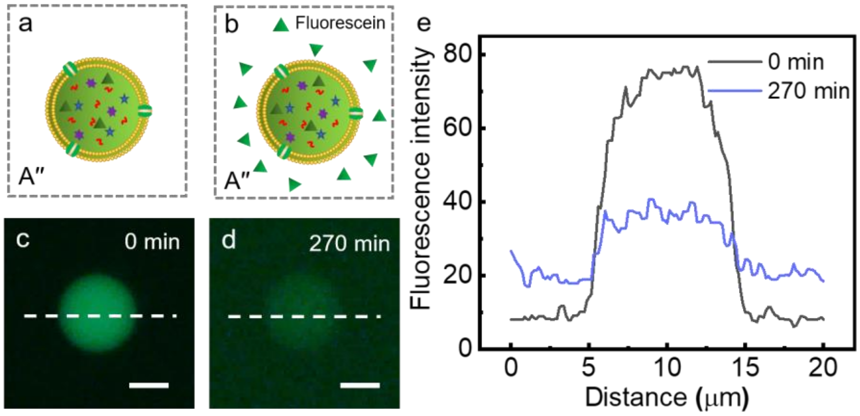

Supplementary Fig. 22. The schematic diagram and microscopy characterization of fluorescein leakage from species A″. (**a, b**) Schematic illustration of fluorescein leakage over time from artificial cell A″ containing pH-responsive molecules, sucrose, G6P, and fluorescein. (**c, d**) The corresponding fluorescence microscope images of A″ at 0 min and 270 min with solution pH 7.2. (**e**) The line profiles of the white dashed line in (c, d). The scale bars were 5 μm. Source data are provided as a Source Data file.

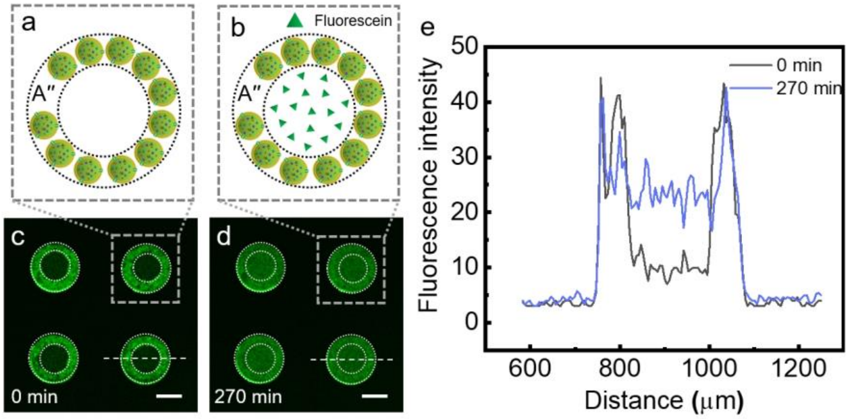

Supplementary Fig. 23. The schematic diagram and microscopy characterization of fluorescein leakage from spatially patterned species A″. **(a, b)** Schematic illustration of fluorescein leakage over time from spatially patterned A″ by magnetic field. **(c, d)** The corresponding fluorescence microscope images of spatially patterned A″ at 0 min and 270 min with solution pH 7.2. **(e)** The line profiles of the white dashed line in (c, d). The scale bars were 200 μm. The scale bars were 5 μm. Source data are provided as a Source Data file.

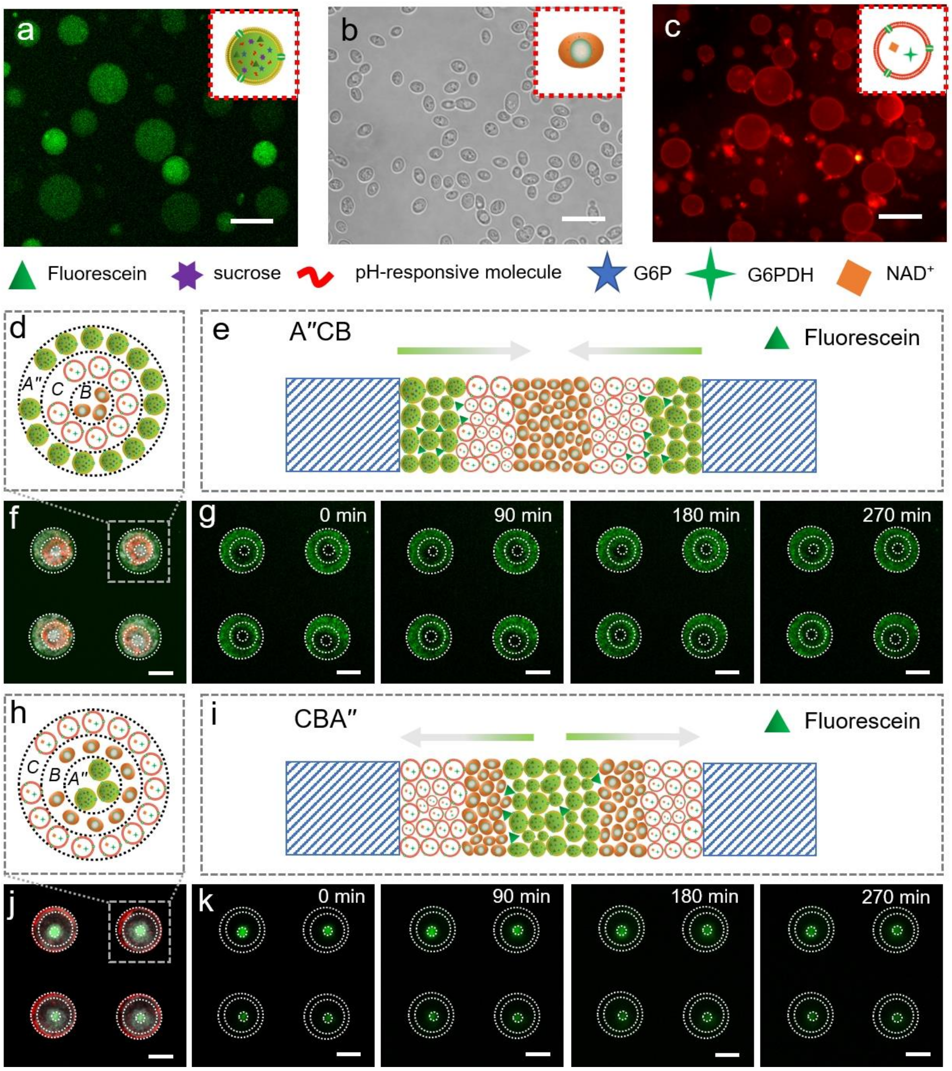

Supplementary Fig. 24. The influence of molecule communications among species in spatially coded communities. (**a**) Species A″ encapsulating fluorescein and pH-responsive molecules. (**b**) *Saccharomyces cerevisiae* (species B). (**c**) Species C labelled with TR DHPE. Schematic diagram of A″CB community with top view (**d**) and side view (**e**). (**f**) Merged image of laser scanning confocal images of A″CB community taken with red and green channels, and white field. (**g**) Laser scanning confocal microscopy images of A″CB community with green channel over time. Schematic diagram of CBA″ community with top view (**h**) and side view (**i**). (**j**) Merged image of laser scanning confocal images of CBA″ community taken with red and green channels, and white field. (**k**) Laser scanning confocal microscopy images of CBA″ community with green channel over time. The scale bars were 20 μm in a, b and c, and 100 μm in f, g, j, and k. G6PDH and G6P are the abbreviations of glucose-6-phosphate dehydrogenase and glucose-6-phosphate, respectively. NAD^+ is the abbreviations of nicotinamide adenine dinucleotide.
