# Supplementary material for: Regulation of species metabolism in synthetic community systems by environmental pH oscillations
Source: Nat Commun. 2023 Nov 18;14:7507. doi: 10.1038/s41467-023-43398-6 (PMC10657449; doi:10.1038/s41467-023-43398-6)
Supplement: Supplementary file 3 — Reporting Summary [file 41467_2023_43398_MOESM3_ESM.pdf]

nature portfolio

Corresponding author(s): Xiaojun Han

Last updated by author(s): Nov 2, 2023

Reporting Summary

Nature Portfolio wishes to improve the reproducibility of the work that we publish. This form provides structure for consistency and transparency in reporting. For further information on Nature Portfolio policies, see our Editorial Policies and the Editorial Policy Checklist.

Statistics

For all statistical analyses, confirm that the following items are present in the figure legend, table legend, main text, or Methods section.

n/a	Confirmed	
☐	☒	The exact sample size (*n*) for each experimental group/condition, given as a discrete number and unit of measurement
☐	☒	A statement on whether measurements were taken from distinct samples or whether the same sample was measured repeatedly
☐	☒	The statistical test(s) used AND whether they are one- or two-sided<br>*Only common tests should be described solely by name; describe more complex techniques in the Methods section.*
☒	☐	A description of all covariates tested
☒	☐	A description of any assumptions or corrections, such as tests of normality and adjustment for multiple comparisons
☐	☒	A full description of the statistical parameters including central tendency (e.g. means) or other basic estimates (e.g. regression coefficient) AND variation (e.g. standard deviation) or associated estimates of uncertainty (e.g. confidence intervals)
☐	☒	For null hypothesis testing, the test statistic (e.g. *F*, *t*, *r*) with confidence intervals, effect sizes, degrees of freedom and *P* value noted<br>*Give P values as exact values whenever suitable.*
☒	☐	For Bayesian analysis, information on the choice of priors and Markov chain Monte Carlo settings
☒	☐	For hierarchical and complex designs, identification of the appropriate level for tests and full reporting of outcomes
☒	☐	Estimates of effect sizes (e.g. Cohen's *d*, Pearson's *r*), indicating how they were calculated

Our web collection on statistics for biologists contains articles on many of the points above.

Software and code

Policy information about availability of computer code

Data collection	Fluorescence microscope image data were collected using fluorecence microscope (Olympus X73, Japan) and laser confocal microscope (Olympus FV 3000,Japan). The pH of the solution was monitored using a precise micro pH meter (Mettler Toledo, Switzerland). Flow cytometry (BD LSRFortessa,USA) was used to test population density. The fluorescence change of species C was monitored by a fluorescence spectrometer (PerkinElmer, USA)
Data analysis	Fluorescence microscope images were analyzed by CelSens software and Image J. The data of flow cytometry was analyzed by FlowJo 7.6. Data process was conducted using Origin 2018 and Office 2019.

For manuscripts utilizing custom algorithms or software that are central to the research but not yet described in published literature, software must be made available to editors and reviewers. We strongly encourage code deposition in a community repository (e.g. GitHub). See the Nature Portfolio guidelines for submitting code & software for further information.

Data

Policy information about availability of data

All manuscripts must include a data availability statement. This statement should provide the following information, where applicable:

- Accession codes, unique identifiers, or web links for publicly available datasets
- A description of any restrictions on data availability
- For clinical datasets or third party data, please ensure that the statement adheres to our policy

The source data underlying Fig. 1e, 1f, 2b, 2c, 2d, 3b, 3c, 3e, 4a, 4c, 4d, 4f, 4g, 4i and Supplementary Fig. 2, 3, 4, 7, 9, 10b, 10c, 10d, 10e, 11, 13, 15, 16, 20b, 20d,

20f, 22e, 23e are provided as a Source Data file.

Research involving human participants, their data, or biological material

Policy information about studies with human participants or human data. See also policy information about sex, gender (identity/presentation), and sexual orientation and race, ethnicity and racism.

Reporting on sex and gender	N/A
Reporting on race, ethnicity, or other socially relevant groupings	N/A
Population characteristics	N/A
Recruitment	N/A
Ethics oversight	N/A

Note that full information on the approval of the study protocol must also be provided in the manuscript.

Field-specific reporting

Please select the one below that is the best fit for your research. If you are not sure, read the appropriate sections before making your selection.

[x] Life sciences [ ] Behavioural & social sciences [ ] Ecological, evolutionary & environmental sciences

For a reference copy of the document with all sections, see nature.com/documents/nr-reporting-summary-flat.pdf

Life sciences study design

All studies must disclose on these points even when the disclosure is negative.

Sample size	For the communication among spatially coded three-specie-community experiments, the fluorescence intensities of at least 10 regions per independent experiment were measured.
Data exclusions	No such exclusion in this paper.
Replication	We have confirmed that at least three independent attempts at replications were successful.
Randomization	The allocation was randomization.
Blinding	No blinding tests were needed since only yeasts were used in this paper.

Reporting for specific materials, systems and methods

We require information from authors about some types of materials, experimental systems and methods used in many studies. Here, indicate whether each material, system or method listed is relevant to your study. If you are not sure if a list item applies to your research, read the appropriate section before selecting a response.

Materials & experimental systems

n/a	Involved in the study	
[x]	[ ]	Antibodies
[ ]	[x]	Eukaryotic cell lines
[x]	[ ]	Palaeontology and archaeology
[ ]	[x]	Animals and other organisms
[x]	[ ]	Clinical data
[x]	[ ]	Dual use research of concern
[x]	[ ]	Plants

Methods

n/a	Involved in the study	
[x]	[ ]	ChIP-seq
[ ]	[x]	Flow cytometry
[x]	[ ]	MRI-based neuroimaging

Eukaryotic cell lines

Policy information about cell lines and Sex and Gender in Research

Cell line source(s)	Yeasts were obtained from Mingzhou Biotechnology Co, Ltd (B82191, China)
Authentication	The cell line was authenticated by the supplier.
Mycoplasma contamination	Mycoplasma contamination was not tested. Yeasts were used to study the consumption of sucrose.
Commonly misidentified lines (See ICLAC register)	No commonly misidentified lines were used in this study.

Animals and other research organisms

Policy information about studies involving animals; ARRIVE guidelines recommended for reporting animal research, and Sex and Gender in Research

Laboratory animals	The study did not involve laboratory animals.
Wild animals	No wild animals were used in the study
Reporting on sex	The study did not include any research involving humans or animals.
Field-collected samples	No field collected samples were used in the study.
Ethics oversight	The study did not include any research involving humans or animals.

Note that full information on the approval of the study protocol must also be provided in the manuscript.

Plants

Seed stocks	N/A
Novel plant genotypes	N/A
Authentication	N/A

Flow Cytometry

Plots

Confirm that:

- [x] The axis labels state the marker and fluorochrome used (e.g. CD4-FITC).
- [x] The axis scales are clearly visible. Include numbers along axes only for bottom left plot of group (a 'group' is an analysis of identical markers).
- [x] All plots are contour plots with outliers or pseudocolor plots.
- [x] A numerical value for number of cells or percentage (with statistics) is provided.

Methodology

Sample preparation	The culture medium was removed by centrifuging the yeast solution at 800 × g, and the yeasts were dispersed using gadobutrol solution. The supernatant was removed by centrifuging again to remove the remaining culture medium.
Instrument	BD LSRFortessa
Software	FlowJo 7.6
Cell population abundance	The abundance of yeasts group in the sorted score was 10000. The purity of the sample was investigated through microscopic observation.
Gating strategy	The starting yeasts were selected by the FSC/SSC gates to find reasonable size yeasts with internal complexity. We choose

yeasts with positive FSC and positive SSC, and discarded cells with extremely high SSC or FSC. For the Live/dead assay (FDA/PI), the fluorescent unlabeled yeast was used as the control group, and the fluorescent stained yeast was used as the experimental group.

☒ Tick this box to confirm that a figure exemplifying the gating strategy is provided in the Supplementary Information.
